# Exploring use of activity monitors for patients with obesity during weight-loss treatment - a qualitative study

**DOI:** 10.1186/s13102-021-00253-9

**Published:** 2021-03-17

**Authors:** Ingrid S. Følling, Line M. Oldervoll, Christina Hilmarsen, Ellen M. I. Ersfjord

**Affiliations:** 1 Centre for Obesity Research, Department of Surgery, Forsyningssenteret, St. Olavs Hospital, Trondheim, 7006 Norway; 2 Department of Public Health and Nursing, Faculty of Medicine and Health Sciences, Norwegian University of Science and Technology, Trondheim, 7491 Norway; 3 Centre for Crisis Psychology, Faculty of Psychology, University of Bergen, Bergen, 5020 Norway; 4 Department of Circulation and Medical Imaging Faculty of Medicine and Health Sciences, Norwegian University of Science and Technology, Trondheim, 7491 Norway; 5 Centre for eHealth, University of Agder, Campus Grimstad, 4879 Norway

**Keywords:** Obesity, Activity monitors, Weight-loss programme, Health habits, Qualitative research

## Abstract

**Background:**

Obesity is a major health concern in western countries. In Norway, patients with obesity can attend weight-loss programmes, which focus on changes in dietary and physical activity habits. Use of self-monitoring is advocated when changing dietary and physical activity habits for adults with obesity. This study aimed to explore the experiences of patients with obesity who used activity monitors while attending a weight-loss programme.

**Methods:**

Patients with body mass index (BMI) > 35 kg/m^2^ with weight related comorbidities or a BMI > 40 kg/m^2^ referred to an intermittent weight-loss programme were recruited into this study. They were introduced to one of three different activity monitors, Fitbit Zip™, Mio Fuse™, or Mio Slice™. Semi-structured interviews were performed with patients six months into the weight-loss programme. Thematic analysis was applied when analysing the data.

**Results:**

Of the 29 informants (aged 21 to 66 years) interviewed, 59% were female. Their experience with activity monitors was related to their adherence to the weight-loss programme. Two main themes emerged from the informants stories: 1. “Activity monitors visualize proof of effort or failure to change health habits”. 2. “Activity monitors act as a positive or negative enforcer when incorporating change”.

**Conclusions:**

Using activity monitors either strengthens or undermines patients’ attempts to change health habits when attending a weight-loss program. Our findings suggest a need for more individualized weight-loss programmes for patients with obesity.

**Supplementary Information:**

The online version contains supplementary material available at 10.1186/s13102-021-00253-9.

## Background

The worldwide prevalence of people with obesity almost tripled from 1975 to 2016, and  in 2016 more than 650 million adults were considered to be affected by obesity [1]. Obesity is a complex, multifactorial condition, with genetic, epigenetic, behavioral, socioeconomic, and environmental origins [2]. To reduce obesity, weight-loss programmes promote changes in diet and physical activity [3]. Weight-loss programmes for adults with obesity can lead to clinically important weight loss (5% of baseline weight) [4]. Thus, achieving weight loss is challenging and weight maintenance is hard [3, 5]. To help modify changes in diet and physical activity for adults with obesity, the use of activity monitors as a means of self-monitoring is advocated by the World Health Organization and in international policy [6].

There has been scepticism regarding the use of self-monitoring technologies to support individuals’ behavioural change [7]. Recording and measuring behaviours or bodily functions can be reductionist, disempowering and act disciplining rather than motivational [8]. Nevertheless, the purpose of self-monitoring is to increase awareness of the body and one’s health behaviour [9]. Studies on individuals who used activity monitors have found that it may improve health management [10, 11] and that it strengthens reflections regarding improvement of the self [12]. It was also found that adding activity monitors to interventions for adults with overweight or obesity may increase physical activity [13]. A recently published systematic review and meta-analysis of randomized controlled trials (RCT) found that physical activity monitors had a moderate and significant effect on reduction in body weight and waist circumference, and a large and significant effect on reduction in body mass index (BMI) [14]. However, participants in such studies have been recruited from the general population and may, to a high degree, have been early adopters of technology or already highly motivated to make changes in their diet or increase physical activity level [15]. Qualitative studies on how individuals with overweight or obesity experience self-monitoring have often used subsamples from RCTs [16], which suggest that these informants are more engaged in self-monitoring than if they had been recruited in another way (i.e., a random sample of patients in a weight-loss programme). To date, activity monitors have not been used as a supplement in conventional treatment for patients with obesity in Norway. Activity monitors should not be introduced without knowing patients experiences regarding the use during treatment like weight-loss programmes. Considering the complexity of obesity [2] and that weight-loss is challenging [3], a qualitatively approach exploring patients’ experiences is suitable. Acknowledging the research gap described above, the present study aimed to explore how patients with severe obesity experience using activity monitors while attending an intermittent weight loss program.

## Methods

### Design

This study employed a qualitative approach with semi-structured in-depth interviews embedded in a prospective observational pilot study.

### Setting

#### The weight-loss programme

The Obesity Clinic at St. Olavs Hospital, Trondheim, Norway, referred patients to a standard conventional treatment in an intermittent weight-loss programme at LHL-klinikkene Røros (LHL-R). Criteria for being referred were a body mass index (BMI) > 40 kg/m^2^ or a BMI > 35 kg/m^2^ with weight related comorbidities i.e. type 2 diabetes, hypertension, dyslipidemia, cardiovascular disease, stroke, sleep apnoea, gallbladder disease, hyperuricemia and gout, and osteoarthritis [17]. They also had to have no serious psychiatric diseases and be able to attend group-based activities.

The weight-loss programme took place over a period of two years (October 2016–October 2018) and patients had four intermittent stays (approximately every 5–6 month) at LHL-R, with each stay lasting three weeks. A multidisciplinary team consisting of a physician, dietician, physiotherapist, occupational therapist, nurse and psychiatric nurse trained in behavioural cognitive therapy (BCT) were responsible for the programme. The programme was based on current guidelines for the management of overweight and obesity in adults [18].

The weight-loss programme included physical activity, dietary sessions and BCT, from Monday to Friday every week during each stay. The physical activity part included two hours of theory in plenary and 31 hours of practical sessions. The physical activity programme offered various activities, focusing on being physically active through endurance and strength exercise. Dietary advice was offered in a one-hour lecture and four hours of group work, focusing on calories and nutrition, two hours of examining the ingredients and nutrition of groceries, and four hours of cooking. Dietary sessions focused on planning and preparing meals, as well as awareness of, and ways to modify, eating behaviour. Personalized meal plans and dietary advice targeted a calorie reduction of approximately 600 Kilocalories a day. Individual calorie restricted diets were based on the given estimate for resting metabolic rate. BCT included three hours of plenary sessions, four hours group work, and individual sessions. BCT was aimed at goal setting and increased awareness of maladaptive cognitions that contribute to the maintenance of emotional distress and problematic eating behaviour [19]. Topics covered were expectations towards attending the weight-loss programme, motivation and conflicting interests, excuses, compensatory strategies and foundation for change. In addition, each participant’s body composition was assessed using a bioelectrical impedance scale. Cardiovascular and metabolic risks were assessed using blood chemistry analysis, and physiology assessed using cardiorespiratory fitness test and blood pressure measurement. The team discussed test results with each participant individually.

For the home periods between the intermittent stays, patients had individualized programmes they were encouraged to follow. These programmes were worked out together with personnel at LHL-R. Each patient had a primary contact from the personnel who performed an individual health conversation at the beginning at the end of each intermittent stay. In these conversations, goals were discussed and set and different issues related to weight-loss and/or other life circumstances was talked about. Aims for their home period were written down in the conversation at the end of each stay. Some got individualized activity programmes, and/or some got dietary plans for their home period. It could also be other issues they had to solve during home periods, i.e. financial problems, emotional or family issues that was seen as barriers in the weight-loss process.

#### Activity monitors

Activity monitors are normally not used in the standard conventional treatment in weight-loss programmes. Thus, for research purpose, three different activity monitors were tested for feasibility. 1, The FitBit Zip™ is a clip-on step counter that records and displays distance and estimates total energy consumption. 2. The Mio Fuse™ and 3. The Mio Slice™ are wristbands with integrated optical heart rate sensors, recording and displaying heart rate, and calorie consumption and sleep hours. The Mio devices held a population-based algorithm calculating heart rate variability into Personal Activity Intelligence points (PAI) [20].

All three activity monitors display “time to move” reminders. When reaching the recommended 10.000 daily steps or 100 weekly PAI, the activity monitors displayed smiley faces and apps changed colour.

Activity monitors and respective mobile software applications were set-up for all patients, device pairing completed, and troubleshooting handled with help from personnel. Patients were encouraged to use the monitors all the time when they were awake, and at least during planned physical activity. The activity monitors were a voluntary supplement so they were free to stop using the activity monitors whenever they wanted. For home periods, all patients were encouraged to reach 10,000 steps per day (Zip users), or 100 PAIs per week (Fuse / Slice).

### Recruitment

All 56 patients who entered their first stay at LHL-R were included in a larger prospective observational pilot study and thereby introduced to one of the three different activity monitors (Fitbit Zip™, Mio Fuse™, or Mio Slice™).

During the patients’ second stay at LHL-R, a random sample was recruited for this study. The team at LHL-R helped with the recruitment process. They informed the patients that participation in the study would not affect their participation in the weight-loss programme. Recruitment continued until saturation was met. Of the 56 patients, 31 were asked to be interviewed, two declined, resulting in 29 informants for this study.

### Interviews

Interviews were conducted between February and July 2017 at patients’ second stay at LHL-R. The third author performed the interviews, which had a mean duration time of 45 min. An audio recorder was used and field notes were made during each interview. Given the exploratory nature of our study, a descriptive approach was most appropriate to elucidate insights in the patients’ experiences when they attended a weight-loss programme.

A semi-structured interview guide was employed (Supplement 1), the main question being “What are your thoughts about how the activity monitor affects your weight-loss?” Follow-up questions and probes were used to clarify and explore what they considered important, such as: “How do you experience the activity monitor?” “What did you like or dislike about the activity monitor?”

### Ethics

The study was approved by the Regional Committee for Medical and Health research in central Norway (REK 2016/833). All informants received oral and written information about the study. They signed an informed consent form prior to the interview.

### Data analysis

All interviews were transcribed verbatim and analyzed together with the field notes. The data was analysed thematically in six steps [21]. In the first step of the analysis, transcripts and field notes were read to obtain an overall impression of the material. A summary of transcripts and a list of preliminary themes that occurred was written. Second, all meaning units were extracted and sorted into codes. In the search for themes were all codes relevant to the aim discussed by all authors in the third step. The co-authors read three interviews each and the summary prior to the discussion. In the fourth step, the themes were reviewed, checking that each theme worked in relation to the codes and related data. Some themes were discarded, and some codes were revised returning to steps two and three. In the fifth step, all authors defined and named the themes after exploring: *“what did this theme tell us?”* and *“how did each story fit the overall story of the data?”* Naming the themes finalized this step. In the sixth and final step, the results were written, including quotes that highlight informants’ stories. The authors met repeatedly between October 2017 and March 2018, discussing themes and possible underlying patterns in the data. All quotes were translated from Norwegian into English. Procedural rigour for the study was established by the range of expertise of the authors, including a nurse with experience in qualitative studies, two physiotherapist (one working clinical with patients with obesity and one researcher) and a social anthropologist with experience in qualitative studies. Analytical rigour was ensured by that the interviewer reviewed the transcripts, and all authors were involved in the development of the coding and themes. There were reflections with regard to each author’s background and experiences and how this could affect data, and the results were checked regarding prejudices or expectations.

The COREQ 32-ithem checklist designed for reporting qualitative research [22] were used to report important aspects of the research team, analysis and interpretation (Supplement 2).

## Results

The 29 informants were all Caucasian, aged between 21 to 66 years, with 59% being females (Table 1). Most informants continued to use activity monitors at six months; however, the majority had changed to a different activity monitor. Reasons for changing to a different monitor included size of wristband, being too small or tight, or being too complicated.

**Table 1:** Informants characteristics

Characteristics	Total (***N*** = 29)
**Sociodemographic variables**	**N (%)**
Gender	
*Females*	17 (59)
*Males*	12 (41)
Age	
*18–29*	3 (10)
*30–39*	5 (17)
*40–49*	12 (42)
*50–59*	7 (24)
*> 60*	2 (7)
Civil status	
*Single/Separated*	18 (62)
*Partner/Married*	11 (38)
Highest level of education	
*Nine years or less of school*	5 (17)
*More than nine years of school*	13 (45)
*Bachelor degree or higher*	11 (38)
Work status	
*Student*	2 (7)
*Working*	15 (51)
*On disability leave*	12 (42)
Weight-related comorbidities	
*≤ 1*	4 (14)
*2–3*	21 (72)
*≥ 4*	4 (14)
Use of activity monitors at six moths	
*Continued with the activity monitors given to them*	10 (34)
*Changed to a preferable activity monitors of their own*	17 (59)
*Not wanting to use activity monitors*	2 (7)

Two main themes emerged from the informants’ stories. In general, descriptions of their experience with activity monitors were aligned with their adherence (or lack thereof) to the weight-loss programme.

### Activity monitors visualize proof of effort or highlights failure to change health habits

In about two thirds of the informants’ stories, the activity monitors acted as proof of effort in the weight-loss programme. The other third of the informants described activity monitors as highlighting their failure to change health habits.

The informants who described activity monitors as evidence of effort emphasized how they themselves had asked professional help to change their health habits. They also said that they had recognized that their health habits needed adjustments both before entering the weight-loss programme and during their first stay at LHL-R.

*“For a long time I have known that I had to do something about my weight, I have exercised too little and I sit all day at work, I have been looking forward to start this change for myself. I think this time I am going to manage it, I have tried before, but then my goals were too high.” Female 30-39 years*

They described finding joy in experiencing new activities and pushing their own boundaries during the stays at LHL-R. Physical activity was mentioned as an investment in their bodies and health.

*“Here (LHL-R) we have tried out a lot of different activities, and after a workout I feel enjoyment over that my body is sweat and my muscles are sore. I never thought I was going to say that, but it actually feels good and I know that is what my body needs. I am doing this for my health.” Male 50-59 years*

For them, the activity monitor acted as evidence of their effort when displaying the regularity of the physical activity, as well as the distance covered and duration of the activity.

*“On the watch, I can see that I have been working hard, so my mind and motivation was better in that regard. There was less focus on my pain, and more on having achieved something good for myself.” Male 20–29 years*

Connecting the numbers with their own perceptions, they described how their bodies now responded differently compared to six months earlier when they started the weight-loss programme. When checking the activity monitors’ physical activity log, they saw how they gradually were able to walk the same route in less time.

*“I have to walk to get my job done so I use the monitor to look to see how far I have come when it is time for lunch. With more walking I become faster, and I get better time and then I will have more spare time. I'm not as tired after my work-walk anymore as I was before, but I'm 40 kilos less than I was before.”  Male 50-59 years*

They talked about how they had modified pre-set activity targets and adjusted their individual goals. The monitors’ display motivated them to keep up with physical activity and diet. Both accumulated physical activity and calorie consumption on the monitor’s display was used as guidance. The activity monitor was useful for monitoring progress in physical activity. Some told how they felt compelled to reach the recommended steps or activity points.

*“I have a very conscious relationship to the watch. I use it all the time when I exercise, to see how far and how fast I walk. It encourages me to do a little bit more when I see that I am close to the target. When reaching the target for a day I feel more satisfied when I go to bed.” Male 40–49 years*

The informants who described activity monitors as highlighting their failure to change health habits said that their motivation for entering the weight-loss programme was primarily pleasing someone else, for example a next of kin or their general practitioner.

*“It was my general practitioner that told me I had to do something about my weight and sent me to the Obesity Clinic and I did not want any surgery, that is why I am here no to lose weight.”  Male 50-59 years*

These informants did not find the weight-loss programme helpful. They said they had never been physically active or enjoyed physical activity.

*“I have never liked to be physically active, and the physical activity classes at school were the worst. Now I at least try to walk every day, but I am so far from meeting recommendation and I do not need a watch to tell me that also.” Female 50–59 years*

Fear of pain and discomfort related to physical activity was frequently mentioned. They explained they did not manage to meet the goals they had set with the team initially in the weight-loss programme.

*“I have so much pain when I try to walk, so it is impossible for me to reach the recommendation, and it really annoys me that I cannot reached my target.” Female 30–39 years*

In their stories, it was evident that they failed to follow diet and physical activity instructions, and they continued to make unhealthy choices. Low numbers on the activity monitors visualized their failure in following instructions.

*“I walked much less than I thought; I'm not particularly physically active, much less than I should have done. I ended up not using it because I sat so much still; it was not exactly motivating to see that I moved 20 %  of what I should have.” Female 30–39 years*

They talked about how the monitors made them aware of how little they walked compared to the recommendations, and they said they felt bad about this. It was their own responsibility and they were ashamed.

*“I have had a lot of bad conscience for neither eating proper nor being as active as I should. It is not especially motivating to see that I only move like 20 % of what is recommended. The monitor keeps reminding me when I have been sitting too long, adding to my frustration, and that is just making me feel bad that I cannot do better." Female 20–29 years*

They said they were to blame for not being able to change. In addition, they emphasized how every wrong decision, for example choosing a hot dog over fruit for a snack, or choosing the elevator over the stairs, made them feel bad about themselves. The activity monitor evidenced, and reinforced, their sense of failure.

*“The watch was too small, and it was hard to accept. But I changed to another one, but then I was not good enough at reaching the daily steps. In the beginning I thought that maybe it could be a motivation, but it was just the opposite, so I just had to put it away.” Female 40-49 years*

### Activity monitors act as a positive or negative enforcer when incorporating change

All informants said that daily obligations towards family, friends, work and leisure activities did not allow for several hours of physical activity every day when being home from the intermittent stays.

Thus, the informants who saw monitors as proof of their hard work described activity monitors as a source of support when incorporating change into everyday life. They exercised less than when at the intermittent stays, but regularly. By making small but feasible modifications, they were able to incorporate recommendations from the weight-loss program into everyday life. For them, every step and effort counted, and the activity monitors motivated them to continue to follow the instructions from the weight-loss programme to the best of their ability. The monitor also helped in finding ways of becoming more active and planning for activity. One said:

*“Activity monitors strengthen confidence in my ability to follow instructions*. *The constant surveillance of the body imposed by the monitor visualizes ability to change habits as proposed, and that is why it is a support for me in my work of changing health habits.”*

*Male 30-39 years*

The informants who found that activity monitors highlighted their failure to change described how hard it was to live by the instructions from the weight-loss programme. They said that during their intermittent stays at LHL-R, reaching the activity targets was feasible, while at home it was impossible. They described how they struggled to incorporate changes from the weight-loss programme at home. They did not consider adjusting their activity targets to more realistic levels while at home. However, they were overwhelmed by feelings of defeat, as they were unable to manage everything, as they should. In addition, not reaching physical activity targets as programmed in their activity monitor amplified their description of “nothing I do makes a difference anyway”.

*“What I have really missed is the sensation that this change feels okay. It is just hard work. What the team at LHL-R told me to do when I was at the intermittent stay is not helping when I am home in my daily life. The activity monitor was interesting in the beginning, now it just feels like a burden, reminding me of what I should have done but did not do." Female 50–59 years*

## Discussion

The aim of this present study was to explore how patients with obesity experience activity monitors when being treated for overweight or obesity. In our results, after being introduced to activity monitors at their first stay in the weight-loss programme at LHL-R, most informants continued to use activity monitors after six months. However, the majority had replaced the monitor with a more suitable one. The informants’ description of experiences with activity monitors was related to adherence (or lack of) to the weight-loss programme. Two main themes emerged from informants’ stories: *“Activity monitors visualize proof of effort or failure to change health habits”* and *“Activity monitors act as a positive or negative enforcer when incorporating change.”*

### Positive and negative experiences with activity monitors

In our study, about two thirds of informants told that the activity monitor acted as a proof of effort when attending the weight-loss program. These informants talked about the positive effects of being more physically active and enjoying the response of their bodies, and some continued to lose weight after starting at the weight-loss programme. The intention for weight-loss programmes for patients with obesity should always be to create healthier habits [23]. Previous quantitative studies have found that wearable technologies support physical activity interventions to reduce weight, waist circumference and BMI for individuals with overweight and obesity [14]. Some people with overweight and obesity manage to develop new habits, and goal setting and self-tracking can be effective techniques in doing so [24].

The informants with positive experience in our study were more likely to enter the weight-loss programme on their own initiative, whilst those with negative experience had external motivation for attending the weight-loss programme. This is in line with earlier findings that individuals motivated by own needs and desires find it easier to sustain new behaviour [25]. People who enter such programmes on their own initiative may be more ready for change [26]. They may be also more likely to acknowledge their weight and health problems. They could thereby be more prone to adhere to the activity monitor and develop new habits, both within physical activity and diet.

The finding that informants in our study had positive experiences with the activity monitors contributes to other findings that activity monitors can help in the weight-loss process.

However, in our study, one third of the informants expressed that the activity monitor evidenced their failure to change. These informants told they blamed themselves for being unable to change. Other studies have also found that patients may express self-blame for their failure to lose weight [27, 28]. There may be negative psychological consequences of participating in weight-loss programmes, which could exacerbate continuing disparities in obesity, health and well-being [29]. These psychological consequences of participating in weight-loss programmes may include loss of self-efficacy and self-esteem [30], as well as increase in body dissatisfaction and social stigma [31]. Unfortunately, individuals who have successfully initiated a new habit will more often fail than succeed in maintaining the new habit over time [25]. Another qualitative study about changing health habits found that previous attempts to change was a barrier to new attempts to change [32]. As many previous studies have highlighted, weight maintenance is hard, and many people regain weight after participating in weight-loss programmes [3, 5]. In addition, for people struggling with overweight and obesity, the body metabolism reaction is to go back to the highest weight or more [33]. This again could enforce previous failures in weight-loss attempts and may result in more self-blame. In such cases, attending weight-loss programmes could be contraindicative if participation yields yet another failure.

Based on our results, the use of activity monitors and the experiences with it may indicate which patients will adhere to treatment and who will not. The downsides of using activity monitors were more pronounced for patients that did not adhere to treatment. For them, the activity monitors acted as reinforcing previous failure for patients six months into the weigh-loss program. Our findings support the need for more individualized weight-loss programmes for patients with obesity, and thus a thorough screening for treatment. This applies especially for those who do not adhere to weight-loss programmes, focusing on finding other strategies so all patients with obesity can get adequate help based on their individual background and needs. There is a challenge with use of activity monitors in treatment for patients with obesity in regard to predict who will respond positively and who will not. Nonetheless, our results have given some indicators that can be helpful for both clinicians and patients in the assessment of activity monitors as a supplement in treatment like weight-loss programmes.

### Strengths and limitations

The interviews in this study took place at patients’ second stay at LHL-R, six months after introducing activity monitors, and there is no data on the participants’ experiences with the monitors during the total period of two years. Other studies has shown that initial interest in activity monitors is rapidly lost, whereas over a third of individuals purchasing commercially available activity monitors abandon the use within six months [34]. However, in our sample, only a few had abandoned the use within six months, with most having changed to a different monitor.

Regarding the qualitative design of this study, we cannot generalize our findings to all patients with obesity using activity monitor. Even so, we believe our sample to be potentially more representative to adults with obesity than studies with open recruitment on participants with overweight and obesity [16]. We also do not know the experiences of all 56 patients introduced to activity monitors in the weight-loss programme, as 27 were not interviewed. Thus, our study adds new knowledge on the use of activity monitors for patients with obesity who attend weight-loss programmes.

## Conclusion

The results of this study suggest that using an activity monitor can either strengthen or undermine patients with obesity attempts to change health habits. Patients who entered the weight-loss programme on their own initiative responded more positively to the activity monitors than those who did not, thus clinicians could recommend use of activity monitors for such patients. Our findings highlight the need to focus on a selection of patients with obesity that could benefit from attending weight-loss programmes. Further research should investigate the use of activity monitor in patients with obesity over longer time (> 6 months).

## Supplementary Information

**Additional file 1.** Supplement 1. Topic guides for interviews. A semi-structured interview guide with the questions that were asked to the informants in this study.

**Additional file 2.** Supplement 2. COREQ 32-ithem checklist. A checklist reporting important aspects of the research team, analysis and interpretation

## Data Availability

The datasets analysed during the current study are not publicly available due to the security of the anonymity of the participants according to the Regional Committee for Medical and Health Research Ethics in Central Norway regulations, but are available from the corresponding author on reasonable request.

## Abbreviations

BCT: Behavioural Cognitive Therapy
BMI: Body Mass Index
LHL-R: LHL-klinikkene Røros
PAI: Personal Activity Intelligence points
RCT: Randomized Controlled Trials
REK: Regional Committee for Medical and Health research
