# Supplementary material for: Exploring use of activity monitors for patients with obesity during weight-loss treatment - a qualitative study
Source: BMC Sports Sci Med Rehabil. 2021 Mar 17;13:25. doi: 10.1186/s13102-021-00253-9 (PMC7968213; doi:10.1186/s13102-021-00253-9)
Supplement: Supplementary file 1 — Additional file 1. Supplement 1. Topic guides for interviews. A semi-structured interview guide with the questions that were asked to the informants in this study. [file 13102_2021_253_MOESM1_ESM.docx]

# Topic guide for interviews

Interviews are performed by a PhD candidate located in a quiet room with comfortable seating. The interviewer takes notes on topics interesting to follow-up questions, participant's emotional or other reactions during the interview, and any interruptions. Questions are intended to aid the conversation on to the topics of interest. Each interview opens with setting the frame for the conversation and ends with a short summary for clarifying reasons.

### Setting the frame

- Welcoming and expressing gratitude for participating in the study
- About the interviewer: physiotherapist, PhD student, special interest in cardiovascular health and physical activity in persons with severe obesity
- Study scope: learning more about what it takes to be physically active, and what eventually supports/aids becoming more active
- Explaining use of data and anonymization process prior to publication
- Offering reading and commenting on transcript
- Explaining reasons for recording the conversation and asking for permission to record

#### Topic: Social relationships

- First, can you tell me about your life today: education and/or work, family and living situation?
- Has there been any larger changes in respect to family, work, living conditions or other areas the last year? If yes, what are the changes?
- Have you involved anyone in your lifestyle change? Colleagues, family, significant others?
- Can you tell me about any reactions you might have had on your effort trying to make changes to your life?

#### Topic: Health

- Do you experience any health problems, any diseases, any underlying diseases?
- Diseases or weight issues in nearest family
- Can you tell me about when you started to put on weight? How the obesity developed?
- Have you ever got reactions regarding your body or size from your surroundings? (Family, friends, school, colleagues, health care personnel, others )
- What, in your words would be your motivation to try changing lifestyle? What is important for you? (own decision, others opinion)

#### Topic: Previous weight-loss attempts

- Can you tell me about any previous tries to loose weight? Can you share which methods you have tried?
- Have you ever been involved in other treatments regarding your weight? How did they work out for you?

#### Topic: Expectations to current treatment

- Can you tell med about your expectations prior to entering the current treatment?
- Did you expect having to make any changes in your everyday life? Which changes?
- Have you actually made any changes in your everyday life? Which changes and how did you do it?
- Can you tell me of your expectations towards those professionals involved in your treatment?
- Can you share your reasons for entering this treatment and your goals?

#### Topic: Physical activity

- Can you please tell me about your feelings and habits with physical activity from early childhood on?
- Has anything altered in your feelings towards physical activity since you entered the current treatment?
- Can you tell me about your feelings on breaking a sweat, shortness of breath or exhaustion during PA?
- Can you tell me about activities you like or dislike? Has anything changed since you started the current treatment?
- Do you ever feel good during PA, and if so what does it take? (probes might be reaching a goal, looking at a view, winning, competition, mastery, cooperation with others, being part of something and so on)
- What can motivate you to be physically active?
- What can discourage you from being physically active?
- Can you tell me about what it would take for you to be more physically active? (probes might be appointments, solitude/group, obligations, desire, duty, competition, mastery, feeling good and so on)
- Can you tell me about your activity level now compared to before entering the current treatment? Elaborate on why

#### Topic: Wearables

- Can you tell me about your experiences with the wearable?
- Why you chose to use/not to use it?
- Things that worked well/did not work with the wearable
- Technical issues (software, battery capacity, synchronizations and so on)
- Reasons to keep using/abandon the wearable
- If periodically stopped using, what spurred you on to start using it again?
- Previous experiences with other wearables/apps/pen and paper
- Which features would a perfect wearable have to your preferences?

#### Topic: Opinion of treatment effects at this point

- Can you tell me about any treatment effects you have experienced at this point?
- What are your thoughts about how the treatment has/has not worked for you at this point?
- What about your goals for the treatment, are they the same or have they been modified?

#### Topic: The team and health care personnel

- Can you tell me about your relations to the team of professions in this treatment?
- How about other professions you might have met during this treatment?
- Can you tell me about your relations with the professionals at the obesity clinic that referred your to this treatment?
- Can you tell me about your GP: is he/she interested in your treatment and is he/she supportive to your efforts?
- Are there other health care personnel involved and interested in your treatment; is he/she supportive; if no others, should there have been; whom would ideally have been involved and what would they do if you could decide?

### End of interview

- Summary of conversation
- Bring up topics of interest noted during the conversation if the participant has not elaborated during the conversation
- Checking if the interviewer have got things right
- Asking: Are there things we have not talked about that you think would be important to share?
- If emotional reactions during the conversations: follow-up acknowledging them and asking if he/she ha someone to talk to about these things, and if not offer to make contact with a suitable professional in the current treatment
- Stopping the recording and thanking the participant for his/her time and sharing of information
